# 

**Published:** 1895-09

**Authors:** 


### CONTENTS.

COMMUNICATIONS.

What a Dentist Saw in Examining Five Hundred Crania ..................... 417

MONTHLY DIGEST.

Some Principles Relating to Almalgams ..................................... 448

SELECTIONS.

Pental ..................................................................... 452
Formaline as a Preserving and Hardening Fluid for Histological Purposes... 454
The Necessity of a higher Estimation of Tooth and Mouth Hygiene. ........ 454
Guaiacol ................................................................... 456
Delaware's New Medical Law ................................................ 456
Qualified Practitioners in Belgium ......................................... 456

PROCEEDINGS.

Semi-Centennial Meeting of the Mississippi Valley Dental Association. .... 457

COMMENCEMENTS.

Detroit College of Medicine—Dental Department ............................ 458
University of Minnesota—Dental Department ................................ 458
American College of Dental Surgery ........................................ 459
University of Pennsylvania—Department of Dentistry ........................ 459
Northwestern College of Dental Surgery .................................... 460
University of California—College of Dentistry ............................. 461

EDITORIAL.

American Dental Association Meeting ....................................... 461
Meeting of the National Association of Dental Faculties. .................. 463
The National Association of Dental Technics ............................... 464
A Book Reduction .......................................................... 465
Quackery in China ......................................................... 468
Dentists' Relation not Confidential. ...................................... 468
Obituary—John J. R. Patrick, D.D.S., Belleville, Ills. ..................... 465
Necrology ................................................................. 467
Southern Dental Association ............................................... 467

